# Sentinel lymph node biopsy with blue dye after neoadjuvant chemotherapy in initially node negative and positive breast cancer

**DOI:** 10.1186/s12957-025-04082-9

**Published:** 2025-11-10

**Authors:** Muhammer Ergenç, Ahmet Akmercan, Leyla Semiha Şen, Onur Buğdaycı, Zerrin Özgen, Vedat Bayoğlu, Handan Kaya, Bahadır Güllüoğlu, M. Ümit Uğurlu

**Affiliations:** 1 Breast and Endocrine Surgery Unit, Department of General Surgery, Marmara University School of Medicine, Istanbul, Turkey; 2 Department of General Surgery, Marmara University Pendik Training and Research Hospital, Istanbul, Turkey; 3 Department of Radiology, Marmara University School of Medicine, Istanbul, Turkey; 4 Department of Radiation Oncology, Marmara University School of Medicine, Istanbul, Turkey; 5 Division of Medical Oncology, Department of Internal Medicine, Marmara University School of Medicine, Istanbul, Turkey; 6 Department of Pathology, Marmara University School of Medicine, Istanbul, Turkey

**Keywords:** Neoadjuvant chemotherapy, Sentinel lymph node biopsy, Breast cancer, Survival

## Abstract

**Background:**

This study aimed to evaluate the efficacy of sentinel lymph node biopsy (SLNB) using blue dye-only in patients who underwent neoadjuvant chemotherapy (NACT) and the axillary recurrence rate in these patients. Additionally, we sought to investigate the impact of this approach on disease-free survival (DFS) and overall survival (OS).

**Methods:**

Patients who underwent SLNB with blue dye only after receiving NACT for breast cancer between 2013 and 2021 were retrospectively evaluated. The study included patients with invasive breast cancer who were clinically node-negative (cN0) or node-positive (cN1) at admission, received systemic NACT, and converted to clinically node-negative (ycN0) status at restaging.

**Results:**

A total of 185 patients were included in this study; 68 patients with cN0 tumors remained ycN0 after NACT, and 117 patients with cN1 tumors regressed to ycN0 after NACT. The median age of patients was 44 (IQR 38–54) years. Our SLN identification rate was 91% in patients who were cN0 and 88% in those who were cN1 at admission. Overall, SLN was not identified in 20 patients (10.8%). The median follow-up was 59 (IQR, 45–81) months. There were 8 (4.3%) locoregional recurrences (6 breast/chest wall recurrences and 2 axillary recurrences) and 19 (10.3%) distant recurrences. There was no statistically significant difference between the cN0 and cN1 groups regarding locoregional or distant recurrences. No significant differences were observed between the cN0 and cN1 groups in terms of recurrence, DFS (*p* = 0.673), or OS (*p* = 0.610).

**Conclusions:**

Sentinel lymph node biopsy using blue dye only after neoadjuvant chemotherapy demonstrated acceptable identification rates and low axillary recurrence in this cohort. However, this approach should not be regarded as standard practice in patients with initially node-positive breast cancer. These findings may reflect feasibility in selected settings with limited resources or where dual-tracer mapping or targeted axillary dissection is not available. Further prospective studies with standardized axillary marking and longer follow-up are needed to confirm these outcomes.

## Introduction

The extensive utilization of neoadjuvant chemotherapy (NACT) in breast cancer has enabled the reduction of both breast and axillary lymph node surgeries. While evaluation of tumor response in the breast after neoadjuvant therapy is enabled by breast-conserving surgery (BCS), the optimization of sentinel lymph node (SLN) biopsy continues to be debated [1–3].

It is possible to achieve a complete response in the axillary region in approximately 40% of patients with breast cancer who are initially node positive (cN+) after undergoing NACT. However, the use of sentinel lymph node biopsy (SLNB) for staging the axillary region in this population is controversial due to its low identification rate (IR), high false negative rate (FNR), and absence of long-term local recurrence data [4, 5]. Patients with initial node positive status who receive preoperative systemic therapy may experience a false-negative rate exceeding 10% if SLNB is conducted after treatment. This rate can be decreased by marking the lymph nodes that were biopsied to keep track of their removal, using a dual tracer (radioisotope and blue dye), and by removing at least three sentinel nodes through targeted axillary lymph node dissection [6–8].

Due to the restricted availability of radioisotopes and additional equipment costs, single-tracer SLNBs, particularly those using blue dye, are frequently utilized in certain facilities [4, 9, 10].

The aim of this study was to determine the identification rate of SLN using blue dye-only in patients undergoing NACT and recurrence rate in these patients. Additionally, we sought to investigate the impact of this approach to axillary recurrence and survival.

## Materials and methods

This retrospective, observational single-center study was performed at Marmara University Hospital between October 2013 and October 2021.

Data from patients who underwent surgery after receiving NACT for breast cancer were examined. The Marmara University School of Medicine Clinical Research Ethics Committee approved the study (number: 09.2023.1070).

Prior to and following surgery, information was collected from clinical records, pathology, and radiology reports via the hospital’s electronic health records system. The American Joint Committee on Cancer (AJCC) Staging Manual, 8th edition, was used for the clinical and pathological staging of the patients [11].

### Eligibility criteria

The study included patients with invasive breast cancer who had cN0 or cN1 at the time of the preoperative evaluation, who were referred for systemic NACT without the use of lymph node clips. All patients were clinically node negative (ycN0) at restaging. Patients with inflammatory breast cancer, distant metastases, and N2-3 status before NACT and those with clinical node positivity after NACT were excluded from the study. Patients who underwent SLNB using a dual tracer during surgery were also excluded.

Patients were divided into cN0 and cN1 according to the node status at presentation and further categorized as “1–2” (1–2 SLN) and “3 or more” (≥ 3 SLN) according to the number of sentinel lymph nodes in each group and compared.

The primary outcomes of this study were the sentinel lymph node (SLN) identification rate and axillary recurrence rate in patients who were clinically node-negative (cN0) and clinically node-positive (cN1) at baseline, respectively.

The secondary outcomes were as follows: (1) comparison of axillary recurrence rates after SLNB with 1–2 retrieved nodes versus ≥ 3 nodes among patients who were cN1 at presentation; (2) comparison of axillary recurrence rates after SLNB with 1–2 retrieved nodes versus ≥ 3 nodes among patients who were cN0 at presentation; (3) assessment of any locoregional relapse, distant recurrence, or death in patients with cN0 and cN1 disease; and (4) identification of independent predictors of recurrence.

### Systemic treatment

Before initiating NACT, all patients underwent baseline staging of the primary tumor and axilla. Clinical examination was performed to assess palpable nodal disease, followed by dedicated axillary ultrasonography performed by experienced breast radiologists. In cases with suspicious nodal features but without overt radiologic evidence of malignancy, fine-needle aspiration cytology (FNAC) was obtained to confirm metastatic involvement and to classify patients as clinically node-positive (cN1). Axillary clip placement was not routinely performed during the initial period of the study, as targeted axillary dissection had not yet been implemented as standard practice in our institution. The pre-NACT staging findings were documented and later compared with post-treatment imaging to verify nodal downstaging. In this study, cN status at admission refers to the baseline clinical nodal stage assessed before initiation of neoadjuvant chemotherapy (NACT).

Neoadjuvant systemic therapy included doxorubicin and cyclophosphamide for four cycles followed by paclitaxel for four cycles (AC–T). Other schedules of AC followed by a taxane included weekly paclitaxel for 12 weeks or docetaxel every three weeks for four cycles.

For HER2-positive patients, anti-HER2 therapy (trastuzumab ± pertuzumab) was administered in the neoadjuvant setting and was not given concurrently with anthracyclines. Trastuzumab-based therapy was initiated with the taxane phase or, in some patients, as part of TCH/TCHP regimens (docetaxel + carboplatin ± pertuzumab), in accordance with institutional practice and international safety standards. Owing to national access and reimbursement restrictions in Turkey during the early years of the study, dual blockade was available for only a small subset of patients (approximately 8% of the cohort), while the remaining HER2-positive patients received single-agent trastuzumab. Anti-HER2 therapy was continued postoperatively to complete approximately one year of treatment when feasible. Following NACT, the tumor and axillary response were evaluated clinically through physical examination, magnetic resonance imaging (MRI), and (18)F-FDG positron emission tomography–computed tomography (PET–CT) [12, 13].The final pathology results were reviewed by a multidisciplinary team, and adjuvant treatment plans were determined according to postoperative pathology findings. For the purpose of axillary staging, ycN0 status was defined as the absence of suspicious lymph nodes on both preoperative physical examination and dedicated axillary ultrasonography.

### Surgical procedure

The surgical approach (BCS or mastectomy) was determined by a multidisciplinary tumor board, based on tumor size relative to breast volume, radiologic response to neoadjuvant chemotherapy (NACT), and patient preference. Among patients undergoing mastectomy, the majority received immediate breast reconstruction using either autologous tissue or implant-based techniques, performed in collaboration with the plastic surgery team according to patient preference and clinical suitability.

For SLNBs, 5 mL of 1% isosulfan blue was injected into the subareolar lymphatic plexus, followed by gentle massage for five minutes. Excised sentinel lymph nodes were evaluated intraoperatively with frozen sections to detect metastatic disease. Any metastatic focus, including micrometastases and isolated tumor cells, was considered positive.

### Radiation treatment

All patients who underwent breast-conserving surgery (BCS) received adjuvant whole-breast irradiation, which represents the standard of care. Postmastectomy radiotherapy was administered selectively, based on the initial maximal clinical stage and multidisciplinary tumor board recommendations. Although there are still uncertainties regarding radiotherapy after neoadjuvant chemotherapy, indications were primarily based on the initial maximal clinical stage.

In patients who were clinically N(+) at the beginning but pathologically N0 or ypN + and underwent only SLN biopsy and mastectomy, the chest wall and regional lymphatics (including the internal mammary chain depending on risk factors) were irradiated. In cases where breast-conserving surgery was performed, the whole breast ± tumor bed (with a boost), and regional lymph nodes were irradiated. Adjuvant radiotherapy was administered to patients who were initially clinically node-negative (cN0) and pathologically node-negative (ypN0) after NACT and underwent lumpectomy. The treatment included whole breast irradiation with an additional boost to the tumor bed. For cases up to 2020, a 50 Gy/25 fractions ± 10 Gy boost was applied. After the COVID-19 pandemic, the protocol for adjuvant radiotherapy was shifted to a dose of 40.5 Gy in 15 fractions (START B), which became more commonly utilized. For lumpectomy patients who required a boost dose, 48 Gy/15 fractions were applied using a concomitant boost technique [14–17].

### Statistical analysis

As this was a retrospective cohort study, no formal a priori sample size calculation was performed. All eligible patients treated during the study period were included in the analysis.

Descriptive statistics were used to report the clinical and pathological features of the study sample. Continuous variables are expressed as the mean ± SD and median (interquartile range, IQR), while categorical variables are expressed as frequencies with percentages [n (%)]. The Pearson chi-square (χ2) test or Fisher’s exact test was used to compare categorical variables, whereas Student’s t test (for normally distributed data) or the Mann‒Whitney U test (for nonnormally distributed data) was used to compare continuous variables.

The time from surgery until death (for any reason) was defined as the overall survival (OS), and the time from surgery to any recurrence or death (for any reason) was defined as the disease-free survival (DFS). The Kaplan–Meier method was used to calculate the cumulative survival rates, and the log-rank test was used to compare the survival curves. A p-value less than 0.05 was considered indicating statistical significance. Statistical analysis was performed using the SPSS software package (Statistical Package for Social Sciences for Windows version 22.0; Chicago, IL, USA) and Jamovi (The jamovi project (2022), Sydney, Australia, Jamovi Version 2.3, Computer Software, retrieved from https://www.jamovi.org).

## Results

**Fig. 1:**
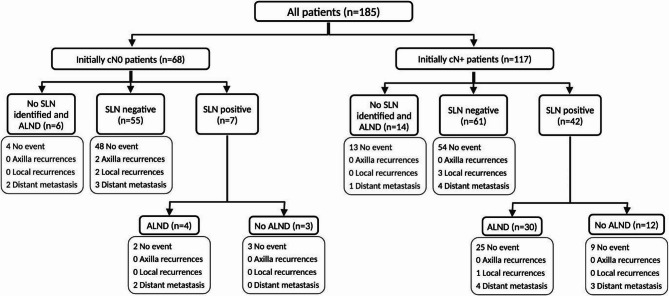
Study flowchart and events of all patients

A total of 185 patients were included in this study; 68 patients were clinically node-negative (cN0) at baseline and remained ycN0 after NACT, while 117 patients who were initially cN1 achieved ycN0 status following NACT (Fig. 1). The median age of the cohorts was 44 years (IQR 38–54). Most patients had ductal histology (*n* = 161, 87%), HR (+)/HER2 (-) subtype (*n* = 83, 44.9%), T2 (*n* = 113, 66.5%) tumors and high (≥ 20%) Ki-67 levels (*n* = 123, 66.5%). HR (+)/HER2 (-) subtype (50.4% vs. 35.3%, *p* = 0.004), cT3/T4 (23.1% vs. 5.9%, *p* = 0.003) tumors, and multifocal/multicentric (49.6% vs. 30.9%, *p* = 0.013) tumors were significantly more common in the cN1 group than in the cN0 group. The clinicopathologic features are detailed in Table 1.

**Table 1:** Clinicopathologic features and outcomes of patients with node-negative and -positive breast cancer at admission

Variables	All Patients *N* = 185	cN0 at admission *n* = 68	cN1 at admission *n* = 117	*p* value
Age (years)	44 (38–54)	46 (39–54)	43 (37–54)	0.315
Menopausal status				0.092
Premenopausal	110 (59.5%)	35 (51.5%)	75 (64.1%)	
Postmenopausal	75 (40.5%)	33 (48.5%)	42 (35.9%)	
Tumor histology				0.216
Ductal	161 (87%)	57 (83.8%)	104 (88.9%)	
Lobular	7 (3.8%)	3 (4.4%)	4 (3.4%)	
Mix of ductal and lobular	3 (1.6%)	0	3 (2.6%)	
Other	14 (7.6%)	8 (11.8%)	6 (5.1%)	
Tumor subtype				0.004
HR (+)/HER2 (-)	83 (44.9%)	24 (35.3%)	59 (50.4%)	
HR (+)/HER2 (+)	39 (21.1%)	13 (19.1%)	26 (22.2%)	
HR (-)/HER2 (+)	27 (14.6%)	10 (14.7%)	17 (14.5%)	
Triple negative	36 (19.5%)	21 (30.9%)	15 (12.8%)	
Ki-67 index				0.642
< 20%	45 (24.3%)	17 (25%)	28 (23.9%)	
≥ 20%	123 (66.5%)	46 (67.6%)	77 (65.8%)	
Unknown	17 (9.2%)	5 (7.4%)	12 (10.3%)	
cT				0.003
cT1	41 (22.2%)	20 (29.4%)	21 (17.9%)	
cT2	113 (61.1%)	44 (64.7%)	69 (59%)	
cT3/4	31 (16.8%)	4 (5.9%)	27 (23.1%)	
Multifocality/multicentricity	79 (42.7%)	21 (30.9%)	58 (49.6%)	0.013
Neoadjuvant chemotherapy regimen				0.610
Anthracycline (AC) + Taxane (T)	96 (51.4%)	33 (48.5%)	63 (53.8%)	
AC → T + Single AntiHER2	54 (29.2%)	20 (29.4%)	34 (29.1%)	
AC → T + Dual AntiHER2	15 (8.1%)	5 (7.4%)	10 (8.5%)	
Other	20 (10.8%)	10 (14.7%)	10 (8.5%)	
Identification rate (n, %)	165 (89.2%)	62 (91.2%)	103 (88%)	0.507
Any recurrence (n, %)	27 (14.6%)	11 (16.2%)	16 (13.7%)	0.642
In-breast/chest wall recurrence (n, %)	6 (3.2%)	2 (2.9%)	4 (3.4%)	0.612
Axillary recurrence (n, %)	2 (1.1%)	2 (2.9%)	0	0.134
Distant recurrence (n, %)	19 (10.3%)	7 (10.3%)	12 (10.3%)	0.994
Death (n, %)	13 (7%)	4 (5.9%)	9 (7.7%)	0.642

Categorical data are expressed as n (%), and continuous data are expressed as medians (IQRs). The unknown category was neglected in the p-value calculation

*HR* Hormone receptor, *HER2* Human epidermal growth factor receptor 2, *cN* Clinical lymph node, *cT* Clinical T stage

In the entire cohort, 15 patients (8.1%) received dual anti-HER2 blockade (trastuzumab + pertuzumab), whereas 54 patients (29.2%) were treated with single-agent trastuzumab in combination with chemotherapy.

Our SLN identification rate was 91.2% in patients with cN0 and 88.0% in those with cN1 at admission. SLN could not be identified in 20 patients (10.8%), all of whom subsequently underwent axillary lymph node dissection.

The median follow-up duration was 59 (IQR, 45–81) months. During the follow-up period, 27 (14.6%) events and 13 (7%) deaths occurred. There were 8 (4.3%) locoregional recurrences (6 breast/chest wall recurrences and 2 axillary recurrences) and 19 (10.3%) distant recurrences. There was no statistically significant difference between the cN0 and cN1 groups regarding locoregional or distant recurrence. (Table 1**).**

The median numbers of SLNs removed using blue dye alone were 4 and 3 for cN0 and cN1, respectively (*p* = 0.174). Additionally, fewer than 3 lymph nodes were removed in 21% of the cN0 group and 20.4% of the cN1 group (*p* = 0.873). Pathologic complete response (pCR), defined as ypT0/is and ypN0, was obtained in 29.2% of all patients. As expected, more pCR was achieved in cN0 patients (32.4%), although a significant difference was not found compared with cN1 patients (27.4%) (*p* = 0.472). Additionally, patients with HR (-)/HER2 (+) and triple-negative molecular subtypes (66.7% and 47.2%, respectively, *p* < 0.001) and high (≥ 20%) Ki-67 levels (35%, *p* = 0.043) had significantly greater pCR rates **(**Table 2**)**.

**Table 2:** Postsurgical characteristics of patients with node-negative and positive breast cancer at admission

Variables	All Patients N = 185	cN0 at admission n = 68	cN1 at admission n = 117	p value
Breast surgery				0.779
BCS	90 (48.6%)	34 (50%)	56 (47.9%)	
Mastectomy	95 (51.4%)	34 (50%)	61 (52.1%)	
Axilla surgery				0.002
SLNB	131 (70.8%)	58 (85.3%)	73 (62.4%)	
SLNB with ALND	34 (18.4%)	4 (5.9%)	30 (25.6%)	
SLNB with no SLN identified and ALND	20 (10.8%)	6 (8.8%)	14 (12%)	
Number of examined SLN				0.873
<3	34 (20.6%)	13 (21%)	21 (20.4%)	
≥3	131 (79.4%)	49 (79%)	82 (79.6%)	
Median	4 (2–5)	4 (2–5)	3 (2–4)	
ypT				0.517
ypTx/Tis	74 (40%)	31 (45.6%)	43 (36.5%)	
ypT1	74 (40%)	23 (33.8%)	51 (43.6%)	
ypT2	26 (14.1%)	9 (13.2%)	17 (14.5%)	
ypT3	11 (5.9%)	5 (7.4%)	6 (5.1%)	
ypN				<0.001
ypN0	125 (67.6%)	60 (88.2%)	65 (55.6%)	
ypNi+	7 (3.8%)	1 (1.5%)	6 (5.1%)	
ypNmi	4 (2.2%)	0	4 (3.4%)	
ypN1	41 (22.2%)	7 (10.3%)	34 (29.1%)	
ypN2	8 (4.3%)	0	8 (6.8%)	
Adjuvant radiotherapy	164 (88.6%)	53 (77.9%)	111 (94.9%)	<0.001
Adjuvant endocrine therapy	132 (71.4%)	40 (58.8%)	92 (78.6%)	0.004

Categorical data are expressed as n (%), and continuous data are expressed as medians (IQRs)

*BCS* Breast-conserving surgery, *SLN* Sentinel lymph node, *SLNB* Sentinel lymph node biopsy, *ALND* Axillary lymph node dissection, *ypN* Pathological lymph node, *ypT* Pathological T stage

Patients with cN0 at admission were categorized as 1–2 SLN and ≥ 3 SLN according to the number of sentinel lymph nodes. Axillary recurrence was detected in 2 patients in the 1–2 SLN group and was statistically significant compared to the ≥ 3 SLN groups (*p* = 0.007) (Table 3**).** Likewise, patients with cN1 at admission were categorized as 1–2 SLN and ≥ 3 SLN according to the number of sentinel lymph nodes. The two groups had no statistically significant difference in recurrence rates and mortality (Table 4**).**

**Table 3:** Disease outcomes after retrieving 1–2 sentinel lymph nodes versus 3 or more nodes in patients with node-negative breast cancer at admission

Variables	cN0 disease at admission				cN0 disease at admission after ALND exclusion			
	Total *n* = 68	1–2 SLN *n* = 15	≥ 3 SLN *n* = 53	*p* value	Total *n* = 58	1–2 SLN *n* = 13	≥ 3 SLN *n* = 45	*p* value
Identification rate (n, %)	62 (91.2%)	13 (86.7%)	49 (92.5%)	0.397				
Any recurrence (n, %)	11 (16.2%)	5 (33.3%)	6 (11.3%)	0.041	7 (12%)	3 (23.1%)	4 (8.9%)	0.167
In-breast/chest wall recurrence (n, %)	2 (2.9%)	0	2 (3.8%)	0.605	2 (3.4%)	0	2 (4.4%)	0.439
Axillary recurrence (n, %)	2 (2.9%)	2 (13.3%)	0	0.046	2 (3.4%)	2 (15.4%)	0	0.007
Distant recurrence (n, %)	7 (10.3%)	3 (20%)	4 (7.5%)	0.161	3 (5.1%)	1 (7.7%)	2 (4.4%)	0.540
Death (n, %)	4 (5.9%)	2 (13.3%)	2 (3.8%)	0.209	4 (6.8%)	1 (7.7%)	1 (2.2%)	0.401

*ALND* Axillary lymph node dissection

**Table 4:** Disease outcomes after retrieving 1–2 sentinel lymph nodes versus 3 or more nodes in patients with node-positive breast cancer at admission

Variables	cN1 disease at admission				cN1 disease at admission after ALND exclusion			
	Total *n* = 117	1–2 SLN *n* = 27	≥ 3 SLN *n* = 90	*p* value	Total *n* = 73	1–2 SLN *n* = 14	≥ 3 SLN *n* = 59	*p* value
Identification rate (n, %)	103 (89.2%)	21 (77.8%)	82 (91.1%)	0.061				
Any recurrence (n, %)	16 (13.7%)	4 (14.8%)	12 (13.3%)	0.844	13 (17.8%)	1 (7.1%)	12 (15.3%)	0.427
In-breast/chest wall recurrence (n, %)	4 (3.4%)	0	4 (4.4%)	0.345	4 (5.4%)	0	4 (5.1%)	0.523
Axillary recurrence (n, %)	0	-	-		-	-	-	
Distant recurrence (n, %)	12 (10.3%)	4 (14.8%)	8 (8.9%)	0.373	7 (9.5%)	1 (7.1%)	6 (10.2%)	0.730
Death (n, %)	9 (7.7%)	3 (11.1%)	6 (6.7%)	0.447	5 (6.8%)	1 (7.1%)	4 (6.8%)	0.667

In the univariate and multivariate Cox regression analyses (Table 5), younger age was independently associated with a higher risk of recurrence (HR 0.94, 95% CI 0.90–0.99, *p* = 0.017). All other clinicopathologic factors, including tumor subtype, Ki-67 index, clinical nodal status, axillary surgery type, and pathologic complete response, were not significantly associated with recurrence.

**Table 5:** Univariate and multivariate Cox regression analyses for predictors of recurrence

Variables	Univariate Analysis		Multivariate Analysis	
	HR (95% CI)	*P* value	HR (95% CI)	*P* value
Age (years)	0.96 (0.93–1.00)	0.069	0.94 (0.90–0.99)	0.017
Tumor subtype				
HR (+)/HER2 (-)	Reference		Reference	
HR (+)/HER2 (+)	0.46 (0.15–1.37)	0.161	0.40 (0.12–1.39)	0.150
HR (-)/HER2 (+)	0.93 (0.31–2.78)	0.890	1.22 (0.34–4.30)	0.761
Triple negative	0.48 (0.14–1.66)	0.249	0.57 (0.15–2.16)	0.413
Ki-67 index				
< 20%	Reference		Reference	
≥ 20%	0.86 (0.33–2.24)	0.757	1.03 (0.35–2.99)	0.963
Clinical nodal status				
cN0	Reference		Reference	
cN1	0.85 (0.39–1.83)	0.675	0.56 (0.24–1.28)	0.168
Axillary surgery				
SLNB	Reference		Reference	
ALND	1.36 (0.62–2.98)	0.436	1.84 (0.73–4.62)	0.193
Pathologic complete response (pCR)				
No	Reference		Reference	
Yes	0.71 (0.30–1.68)	0.434	1.16 (0.41–3.27)	0.785

*Abbreviations:HR* Hazard ratio, *CI* Confidence interval, *SLNB* Sentinel lymph node biopsy, *ALND* Axillary lymph node dissection, *pCR* Pathologic complete response

**Fig. 2:**
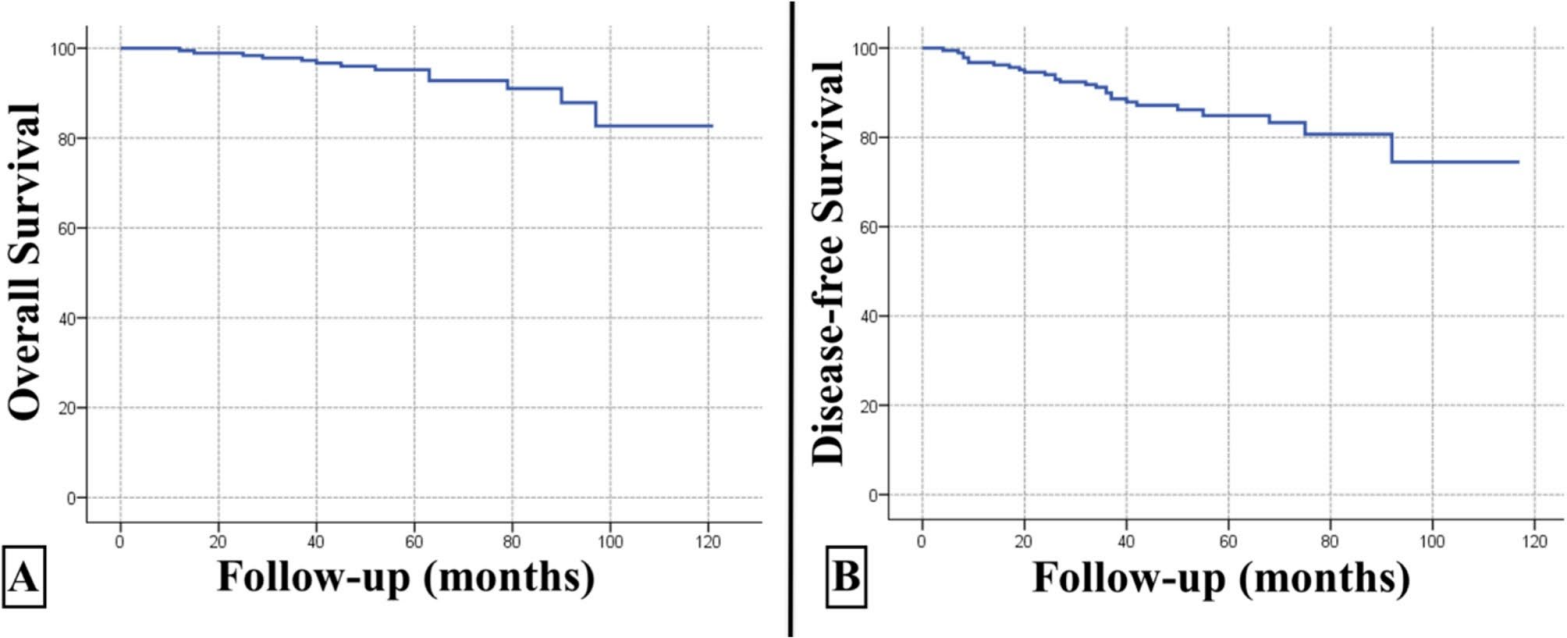
Overall survival (panel **A**) and disease-free survival (panel **B**) curves of all patients

**Fig. 3:**
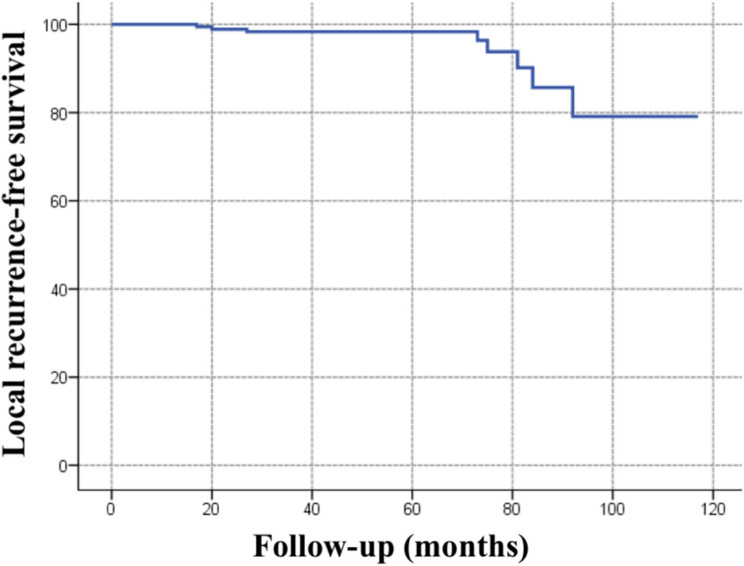
Local recurrence-free survival curves of all patients

**Fig. 4:**
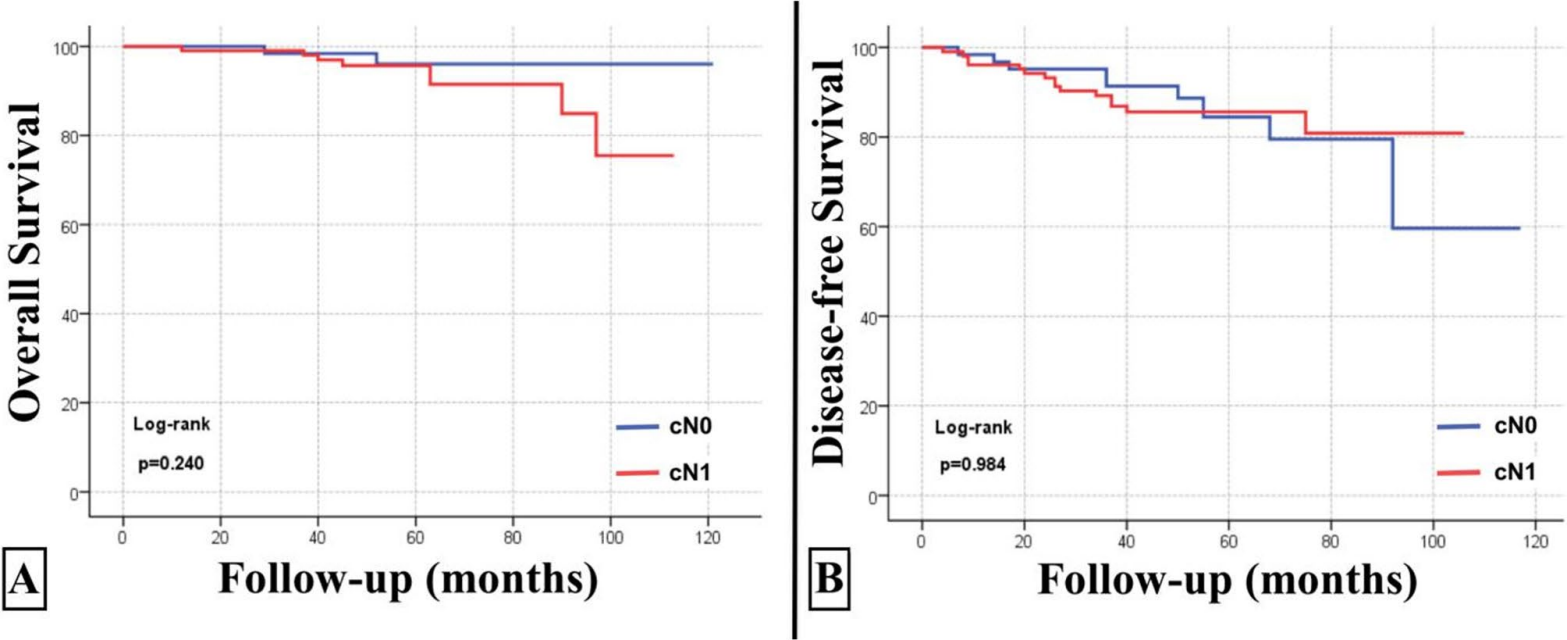
Overall survival (panel **A**) and disease-free survival (panel **B**) curves according to cN status before neoadjuvant chemotherapy

The 5-year OS rate was 95.2% (92.0%−98.5%, 95% CI), and the 5-year DFS rate was 84.9% (79.2%−90.9%, 95% CI) in all patients (Fig. 2). The 5-year locoregional recurrence-free survival (LRFS) rate was 98.3% (96.5%−100%, 95% CI) in all patients **(**Fig. 3**)**. The 5-year OS rate was 94.9% (89.4%−100%, 95% CI), and the 5-year DFS rate was 86.4% (80.2%−93.1%, 95% CI) in the cN0 group. The 5-year OS rate was 95.3% (91.5%−99.4%, 95% CI), and the 5-year DFS rate was 82.3% (71.7%−94.5%, 95% CI) in the cN1 group. There was no statistically significant difference between the groups in terms of OS or DFS (*p* = 0.610 and *p* = 0.673, respectively) **(**Fig. 4**)**.

## Discussion

In this study, we found that the SLN identification rate of blue dye-only method after NACT was 91% in patients with cN0 and 88% in patients with cN1 at admission. SLN could not be identified in 10.8% of patients.

ACOSOG Z1071, SENTINA, and SN FNAC, have investigated the appropriateness of SLNB in patients who clinically convert a cN + axilla to a ycN0 axilla after NACT. These studies have shown that an acceptably low FNR can be achieved in SLNB with the use of the double tracer technique, routine immunohistochemistry, and the acquisition of at least three nodes [3, 7, 10, 18, 19]. The identification rate and false negative rate reported for SLNB in the NSABP B-27 study were comparable to those of standard SLNB without NACT, demonstrating the feasibility of SLNB after NACT [20, 21].

The outcomes of using blue dye only for identification of SLN following NACT are not well known. A study including 34 patients who underwent SLNB after NACT with blue dye only showed that the identification rate of SLN was 85.35% [10]. In a recent study, Cavalcante et al. found that identification rate of SLN using blue dye alone in 100 ycN0 cases who were cN1/2 before NACT was 96% and 5-year disease-free and overall survival were, respectively, 85.9% (95% CI: 74–99.8) and 96.3% (95% CI: 89.4–100) [22]. Our SLN identification rate was 91% in patients with cN0 and 88% in patients with cN1 at admission. We observed axillary recurrence in 2 (1.1%) patients and 5-year OS rate and 5-year DFS rate were 95.2% (92.0%−98.5%, 95% CI) and 84.9% (79.2%−90.9%, 95% CI) respectively.

Galimberti et al. reported outcomes of patients who underwent SLNB independent of targeted axillary dissection (TAD) in patients who were cN + at baseline. Their study found that standard SLNB alone was acceptable in cN + patients with ycN0 after NACT and was not associated with overt axillary disease and a worse prognosis, even in the context of high FNR. A meta-analysis of 27 studies concluded that SLNB following NACT is technically feasible for cN + breast cancer but emphasized the high FNR [1, 23–25].

We could not report FNR because of the design of our study, but the SLN IR and low axillary recurrence were consistent with the literature. Since two patients had axillary recurrence, we could not perform further analysis for axillary recurrence. When the factors associated with any recurrence were evaluated in all patients, we could not obtain statistically meaningful results due to the limited number of recurrence events.

In a review of SLNB after NACT, most of the eligible studies on SLNB after NACT reported similar data to SLNB without NACT and concluded that sentinel node accuracy after NACT was not significantly different from that without NACT; therefore, a clinical trial with extended follow-up is necessary to assess the safety of excluding axillary lymph node dissection in patients with negative sentinel nodes following NACT [20, 26].

In a study investigating SLNB after NACT in patients with stage T1-T3N1M0 breast cancer and positive axillary nodes proven by fine needle aspiration cytology at initial diagnosis, this technique was found to be technically feasible, but it was recommended to be applied according to the molecular subtype of the patients [27]. A meta-analysis of 3,398 patients revealed that performing SLNB following NACT in patients with confirmed positive lymph nodes yielded a satisfactory false negative rate and identification rate, establishing it as a viable alternative to axillary dissection [28]. In a meta-analysis of patients with clinically node-negative breast cancer at baseline after NACT, SLNB was reported to be technically feasible and accurate enough for axillary staging after NACT [29]. According to the literature, the SLN detection and local recurrence rates in this study are acceptable, and it may be feasible to perform SLNB after NACT, even with only blue dye.

Most of the previous studies on SLNB have been performed using radioisotopes, blue dyes, fluorescence, or a combination of two methods. Although we need to include SLNB after NACT, concerns remain regarding the learning curve of the surgical team, the number of lymph nodes removed, the use of single versus dual tracers, and the detection rate and FNR, which may be related to patients’ clinicopathologic characteristics [10, 20]. The use of radioisotopes is limited to authorized institutions, and there are difficulties accessing them; therefore, the method of SLNB varies according to the surgeon and the technical capabilities of the center [20, 30–33]. Our study showed that the results of SLNB with only blue dye are acceptable under obligatory conditions.

This study has several limitations. First, it was a retrospective, single-center analysis with a relatively small sample size, which may limit the generalizability of the findings. Second, axillary lymph node dissection was not routinely performed after SLNB, potentially affecting the accuracy of nodal staging. Nonetheless, the study design reflects real-world clinical practice, which may also be considered a strength. Third, while groups were compared based on initial clinical nodal status, potential confounders—such as imbalances in hormone receptor status—may have influenced the results. Fourth, pre-NACT axillary nodal status was not histologically confirmed in all patients. Although all patients were carefully evaluated with thorough physical examination and axillary ultrasonography, only 27 patients (23.1%) in the clinically node-positive (cN+) group underwent axillary biopsy. This may have resulted in misclassification, particularly among those categorized as cN1. Fifth, the SLN detection rate in our study (89.2%) may reflect limitations of the blue dye-only technique and real-world variability. Although ALND was performed in all patients with failed SLN detection (10.8% of the cohort), the small size of this subgroup limited further analysis. Finally, although univariate analysis revealed associations between recurrence and factors such as age and tumor size, the limited number of events precluded a meaningful multivariate survival analysis. These limitations highlight the need for larger, prospective studies to validate our findings.

## Conclusion

Sentinel lymph node biopsy using blue dye only, following neoadjuvant chemotherapy, achieved acceptable identification rates and low 5-year axillary recurrence in our cohort. However, this approach should not be considered a standard practice for patients with initially node-positive breast cancer. Our findings may reflect feasibility in selected settings with limited resources or where dual-tracer techniques or targeted axillary dissection are not available. Prospective studies incorporating standardized axillary marking and longer follow-up are needed to validate these outcomes.

## Data Availability

The datasets generated during and/or analysed during the current study are available from the corresponding author on reasonable request.

## Abbreviations

AC: Anthracycline and Cyclophosphamide
AC-T: Anthracycline and Cyclophosphamide followed by Taxane
AJCC: American Joint Committee on Cancer
ALND: Axillary Lymph Node Dissection
BCS: Breast-Conserving Surgery
CI: Confidence Interval
cN0: Clinically Node-Negative
cN1: Metastases to movable ipsilateral level I or II axillary lymph node(s)
cN+: Clinically Node-Positive
cT: Clinical Tumor Stage
DFS: Disease-Free Survival
FNR: False-Negative Rate
HER2: Human Epidermal Growth Factor Receptor 2
HR: Hormone Receptor
IQR: Interquartile Range
IR: Identification Rate
LRFS: Locoregional Recurrence-Free Survival
MRI: Magnetic Resonance Imaging
NACT: Neoadjuvant Chemotherapy
OS: Overall Survival
pCR: Pathologic Complete Response
PET-CT: Positron Emission Tomography - Computed Tomography
SD: Standard Deviation
SLN: Sentinel Lymph Node
SLNB: Sentinel Lymph Node Biopsy
START B: Standardisation of Breast Radiotherapy Trial B
TAD: Targeted Axillary Dissection
ypN0: Pathologically Node-Negative after Neoadjuvant Therapy
ypT0/is: Pathologically No Residual Tumor or In Situ Tumor after Neoadjuvant Therapy
ycN0: Clinically Node-Negative at Restaging
